# 1569. Antiretroviral Treatment (ART) Packaging Preferences and Their Impact on Adherence Among People With HIV (PWH): Findings From a Best–Worst Scaling Survey

**DOI:** 10.1093/ofid/ofad500.1404

**Published:** 2023-11-27

**Authors:** Megan Dunbar, Bhumi Gandhi-Patel, Lewis Kopenhafer, Patrick Olsen, Amanda R Mercadante, Nate Way, Kathleen Marie Beusterien

**Affiliations:** Gilead Sciences, Forest City, California; Gilead Sciences, Inc., Foster City, California; Cerner Enviza, North Kansas City, Missouri; Cerner Enviza, North Kansas City, Missouri; Cerner Enviza, North Kansas City, Missouri; Cerner Enviza, North Kansas City, Missouri; Cerner Enviza, North Kansas City, Missouri

## Abstract

**Background:**

ART is highly effective when taken as prescribed, but adherence can be a challenge due to privacy concerns, convenience or stigmatization of HIV (especially among PWH facing health disparities). Blister packs (BPs) may improve medication adherence; however, ART packaging preferences have not been fully explored in PWH. We assessed preferred ART packaging and packaging features perceived to support daily adherence.

**Methods:**

A cross-sectional, online survey was completed by PWH aged 18–65 years living in the US currently on a single-tablet ART regimen. Participants were recruited via patient databases/panels/associations, social media, physician referrals and community centers. The survey elicited preferences for a BP versus a pill bottle. Best–worst scaling (BWS) was used to quantify how PWH prioritized 14 packaging features based on how helpful they were perceived to be for taking daily ART.

**Results:**

The survey population had diverse race/ethnicity and socioeconomic status reflective of US PWH **(Table)**. Of 208 PWH, 91% used pill bottles; 19% were dissatisfied with their current ART packaging and 26% were neither satisfied nor dissatisfied. Less than half (47%) of respondents reported never skipping/missing a medication dose; 25% skipped/missed a dose < 1× a month, and 28% ≥ 1× a month. Overall, 48% of PWH preferred pill bottle packaging, 38% preferred the BP and 15% had no preference. For Black PWH, 40% preferred a BP and 39% preferred a bottle, which differed from preferences in White PWH (35% preferred a BP, 59% preferred a bottle; *P* = 0.04). Among packaging features that PWH found helpful for taking medication as prescribed, the top four were ‘easy to open’, ‘convenient when traveling’, ‘easy to remove one pill at a time’ and ‘discreet’ packaging. PWH who preferred the BP prioritized ‘easy to remove one pill at a time’, ‘convenient when traveling’ and ‘easy to open’ features; those who preferred the bottle prioritized ‘easy to open’, ‘convenient when traveling’ and ‘discreet’ packaging **(Figure)**.

**Figure:**
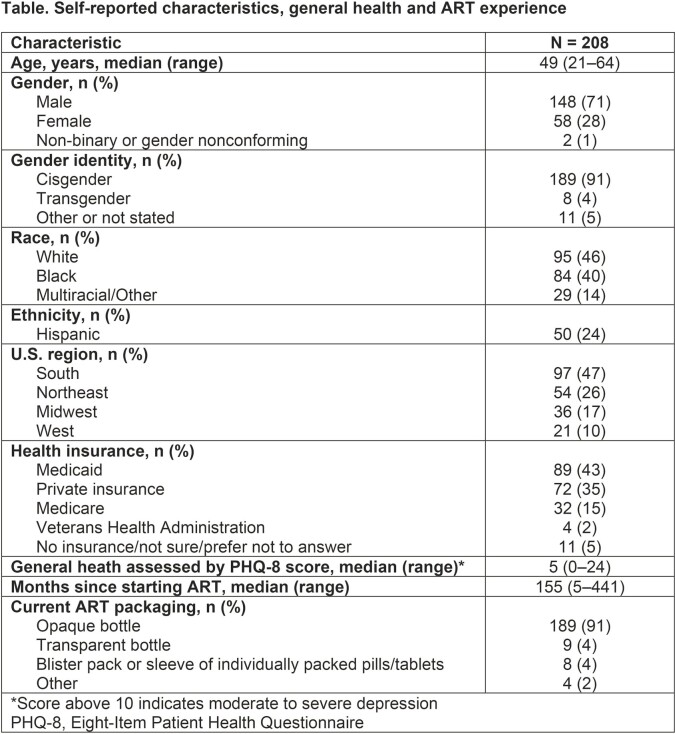

**Figure:**
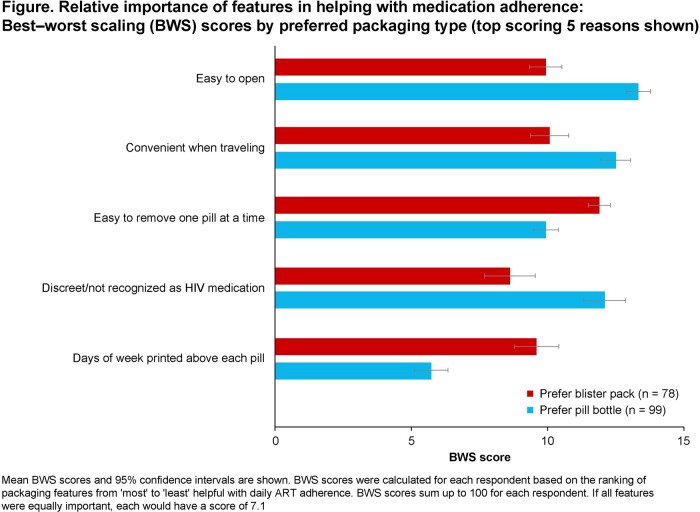

**Conclusion:**

PWH identified packaging features of a BP and/or a pill bottle for ART that may help them adhere to a once-daily treatment regimen. Offering a choice of ART in a BP or bottle may increase convenience and discreet use of HIV medication, helping to address stigma and support adherence.

**Disclosures:**

**Megan Dunbar, PhD**, Gilead: Employment **Bhumi Gandhi-Patel, PharmD**, Gilead: Employment **Kathleen Marie Beusterien, BS, MPH**, Gilead: Employee of Cerner Enviza, which provides consulting services to Gilead

